# Implications of Tioguanine Dosing in IBD Patients with a TPMT Deficiency

**DOI:** 10.3390/metabo13101054

**Published:** 2023-10-06

**Authors:** Debbie S. Deben, Luc J. J. Derijks, Bianca J. C. van den Bosch, Rob H. Creemers, Annick van Nunen, Adriaan A. van Bodegraven, Dennis R. Wong

**Affiliations:** 1 Department of Clinical Pharmacy, Clinical Pharmacology and Toxicology, Zuyderland Medical Centre, 6162 BG Sittard, The Netherlands; d.wong@zuyderland.nl; 2 Department of Clinical Pharmacy and Pharmacology, Máxima Medical Centre, 5504 DB Veldhoven, The Netherlands; 3 Department of Clinical Genetics, Maastricht University Medical Centre (MUMC+), 6229 HX Maastricht, The Netherlands; 4 Department of Gastroenterology, Zuyderland Medical Centre, 6162 BG Sittard, The Netherlands; 5 Department of Gastro-Enterology, Maastricht University Medical Centre (MUMC+), 6229 HX Maastricht, The Netherlands

**Keywords:** thiopurine S-methyl transferase (TPMT), tioguanine, inflammatory bowel disease (IBD), TPMT deficiency

## Abstract

Tioguanine is metabolised by fewer enzymatic steps compared to azathioprine and mercaptopurine, without generating 6-methylmercaptopurine ribonucleotides. However, thiopurine S-methyl transferase (TPMT) plays a role in early toxicity in all thiopurines. We aimed to describe the hazards and opportunities of tioguanine use in inflammatory bowel disease (IBD) patients with aberrant TPMT metabolism and propose preventative measures to safely prescribe tioguanine in these patients. In this retrospective cohort study, all determined *TPMT* genotypes (2016–2021) were evaluated for aberrant metabolism (i.e., intermediate and poor TPMT metabolisers). Subsequently, all IBD patients on tioguanine with aberrant *TPMT* genotypes were evaluated for tioguanine dosages, adverse drug events, lab abnormalities, treatment duration and effectiveness. *TPMT* genotypes were determined in 485 patients, of whom, 50 (10.3%) and 4 patients (0.8%) were intermediate and poor metabolisers, respectively. Of these patients, 12 intermediate and 4 poor TPMT metabolisers had been prescribed tioguanine in varying doses. In one poor TPMT metaboliser, tioguanine 10 mg/day induced delayed pancytopenia. In general, reduced tioguanine dosages of 5 mg/day for intermediate TPMT metabolisers, and 10 mg two-weekly for poor TPMT metabolisers, resulted in a safe, long-term treatment strategy. Diminished or absent TPMT enzyme activity was related with a pharmacokinetic shift of tioguanine metabolism which is associated with relatively late-occurring myelotoxicity in patients on standard tioguanine dose. However, in strongly reduced dose regimens with strict therapeutic drug and safety monitoring, tioguanine treatment remained a safe and effective option in IBD patients with dysfunctional TPMT.

## 1. Introduction

The incidence and prevalence of inflammatory bowel disease (IBD), including Crohn’s disease (CD) and ulcerative colitis (UC), are rising worldwide [1,2,3]. The conventional thiopurine derivatives azathioprine (AZA) and mercaptopurine (MP) remain standard maintenance treatment of IBD. Unfortunately, up to 50% of patients discontinue thiopurine therapy within the first two years of use due to inadequate response or adverse drug events [4,5,6]. Optimization of thiopurine therapy is warranted to maximize treatment success in order to postpone or prevent second-line (expensive) biological treatment or surgery [7,8].

**Figure 1:**
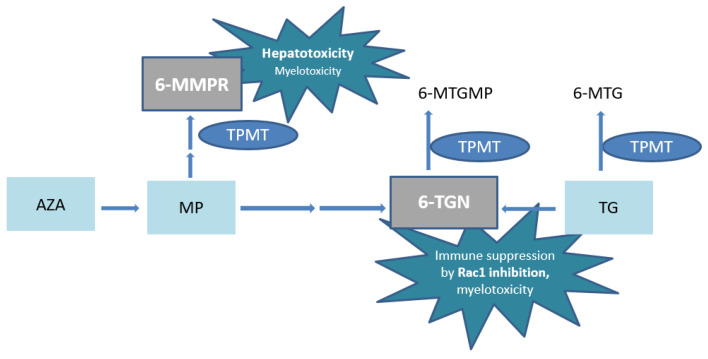
Simplified scheme of thiopurine metabolism. AZA, azathioprine; MP, 6-mercaptopurine; 6-MMPR, 6-methylmercaptopurine ribonucleotides; 6-MTGMP, 6-methylthioguanine monophosphate; TG, tioguanine; 6-MTG, 6-methylthioguanine; TPMT, thiopurine S-methyl transferase; 6-TGN, 6-thioguaninenucleotides; Rac1, Ras-related C3 botulinum toxin substrate 1.

Azathioprine or MP undergoes complex metabolism with generation of the active metabolites 6-thioguanine nucleotides (6-TGN) and the potentially hepatotoxic 6-methylmercaptopurine ribonucleotides (6-MMPR) (Figure 1) [9,10]. The pharmacologically active 6-TGN comprise 6-thioguanine mono–, di– and triphosphates, of which the latter are primarily ascribed to therapeutic effectiveness by inhibition of the small GTPase Ras-related C3 botulinum toxin substrate 1 (Rac1) (Figure 1).

In high concentrations, 6-TGN are also associated with myelotoxicity [11,12]. In addition, extremely high 6-MMPR concentrations have also been associated with myelotoxicity [13]. In conventional thiopurines, thiopurine S-methyl transferase (TPMT) enzyme activity is pivotal in the balance between 6-MMPR and 6-TGN generation and the resulting ratio predicts (hepato-)toxicity and treatment failure [5,14].

Several strategies are being applied in clinical practice to avoid or minimize adverse drug events of thiopurine therapy. One of these strategies is coadministration of the xanthine oxidase inhibitor, allopurinol (100 mg once daily (o.d.)), to a conventional thiopurine in a reduced dosage of 25–33% of the regular thiopurine dosage used for IBD treatment [15]. Combining allopurinol with AZA or MP has proven to be an effective strategy, leading to an altered 6-TGN/6-MMPR ratio by decreasing 6-MMPR formation in favour of 6-TGN. However, addition of allopurinol can also introduce adverse drug events or intolerability.

Tioguanine (TG), an alternative thiopurine derivative, has also been suggested as a simpler strategy for patients who failed AZA or MP therapy [16,17]. Tioguanine had been originally developed as a treatment option for acute leukaemia. Years later, TG was also introduced as a therapeutic agent to prevent rejection of transplanted organs. Notably, decades later, in several countries TG is now also considered as an alternative thiopurine derivative for IBD patients who failed AZA or MP [18]. In multicentre cohort studies, TG was tolerated in up to 80% of patients who previously failed conventional thiopurine therapy [16,19]. In a recent study, TG was proven to be equally effective compared to concomitant use of allopurinol [20]. Recently (i.e., January 2022), TG has been registered for adult IBD patients in the Netherlands (at an indicative dose regimen of 0.3 mg/kg bodyweight o.d., maximum 25 mg/day). Here, approximately 8000 IBD patients are currently on maintenance treatment with TG [21].

The metabolic pathway of TG is less complex with a direct conversion into the pharmacologically active 6-TGN. However, TG is in part also methylated to the inactive 6-methylthioguanine (6-MTG) (Figure 1) [22]. Since no 6-MMPR are formed by TPMT, the risk of (immediate) drug-induced hepatotoxicity is decreased compared to AZA or MP [19]. In addition, a clinically faster onset of action of TG has been reported [23,24]. Whether TG is associated with a higher risk of (long term) hepatotoxicity, particularly nodular regenerative hyperplasia (NRH) of the liver, compared to conventional thiopurines is still debated. The absolute risk seems limited when applying the relatively low doses as advocated in treatment of IBD patients, with dose adaptation if 6-TGN concentrations are exceeding 1000 pmol/8 × 10^8^ red blood cells (RBC) [25,26,27,28].

Despite its simple metabolism, TG may also induce myelotoxicity, even when prescribed in the suggested low dose for IBD [29,30]. In current guidelines, dose adaptations of 50–80% of the registered TG dose are recommended for intermediate TPMT metabolisers, while a 10-fold reduction (0–10%) is advised for poor metabolisers [22,31]. Additionally, pre-emptively *TPMT* testing is recommended for all thiopurines. However, TG dose recommendations are based on dosing schemes for malignancies. For non-malignant conditions, TG dose recommendations for aberrant TPMT metabolism are not yet available.

The aim of this study was to investigate adverse drug events, clinical outcome and optimal dose regimen in IBD patients with an aberrant *TPMT* genotype treated with TG.

## 2. Materials and Methods

### 2.1. Study Design and Patient Population

We conducted a retrospective observational single centre study in Zuyderland Medical Centre, one of the largest referral teaching hospitals for IBD care in the Netherlands. In this centre, *TPMT* genotype assessment prior to initiation of thiopurine therapy for IBD patients has been part of usual care since 2020. Before then, *TPMT* genotypes were also determined in retrospect in case of thiopurine-induced toxicity or following establishment of supratherapeutic 6-TGN concentrations. Data on TPMT enzyme activity were not available.

All *TPMT* genotype results from January 2016 to December 2021 were collected. Next, *TPMT* genotype results were sorted out by the following phenotypes: Normal Metabolisers (NM), defined by *1/*1 genotype (wild type), Intermediate Metabolisers (IM), defined by *1/*2, *1/*3A, *1/*3B, *1/*3C, *1/*8 or *1/*9 (heterozygote variants) and Poor Metabolisers (PM), defined by two of the following null alleles *2, *3A, *3B, *3C, *8, *9 or c.702T > A. Subsequently, electronic medical files of all IM and PM patients were screened for the following inclusion criteria: diagnosis of IBD, age > 18 years and previous or current use of TG.

The diagnosis of IBD (i.e., CD, UC and unclassified IBD (IBD-U)), was based on the standard combination of clinical, biochemical, endoscopic, radiologic and histological findings. Physician global assessment (PGA) was used to define the clinical disease activity status.

In our region, a starting dose of TG 10 mg o.d. is considered to be the standard dose more than the registered bodyweight adapted dosing scheme, whereupon TG dose is optimized guided by TDM.

For analysis, the registered TG dose of 0.3 mg/kg bodyweight o.d., maximum 25 mg/day, was referred to as the standard dose [30]. Tioguanine dosages in this cohort were expressed in daily dose (mg/kg) and daily dose in percentages of registered dose (%).

### 2.2. Data Collection

Demographic, clinical and biochemical data were retrieved from the patient’s medical files using Microsoft Excel, version 2108. Data collection comprised patient and disease characteristics, such as age at time of TG initiation, daily defined TG dose, treatment duration, gender, IBD disease type and previous and current (co)treatment. Furthermore, all available 6-TGN concentrations during TG treatment were also collected.

### 2.3. Thiopurine Metabolite Measurements

The 6-TGN concentrations were determined by the Clinical pharmacological and Toxicological Laboratory of the Department of Clinical Pharmacy of Zuyderland Medical Centre. This analytical determination was routinely performed using a validated assay in erythrocytes based on the Dervieux-Boulieu method [32]. The lower and upper limits of quantification for 6-TGN concentrations were 40 pmol/8 × 10^8^ RBC and 6000 pmol/8 × 10^8^ RBC, respectively.

### 2.4. TPMT Genotype Measurements

All *TPMT* genotypes were determined using Sanger sequence analysis at the Laboratory of Clinical Genetics of the Maastricht University Medical Centre+ (MUMC+). The *TPMT* genotype was routinely determined for the most common variants identified in the Caucasian population (responsible for 95% of the *TPMT* deficient alleles): *2, *3A, *3B and *3C. Within the same analysis, the presence of the rare *8 and *9 variants was also screened for, since the presence of these rare alleles lies within the same fragments of the analysis.

### 2.5. Statistics

All statistical analyses were performed using IBM SPSS Statistics for Windows version 26.0 (IBM Corp., Armonk, NY, USA). Numerical and categorical variables were presented as median and inter-quartile range (IQR; 25th–75th percentiles) and number of patients (%), respectively.

### 2.6. Ethical Considerations

The medical research ethics committee (MREC) of Zuyderland Medical Centre approved this study (number Z2022090). The research protocol is in line with ethical guidelines of the Declaration of Helsinki (2013). Because data were anonymously provided, written informed consent for data analysis and reporting was waived by the MREC.

## 3. Results

### 3.1. Patient Characteristics

**Figure 2:**
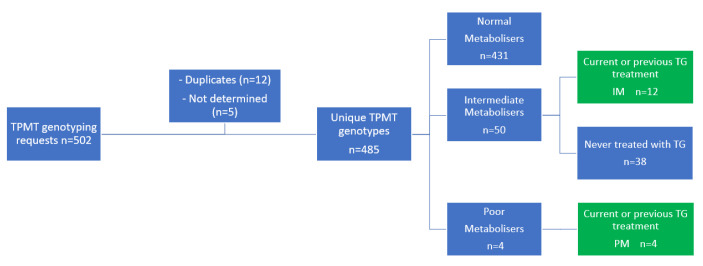
Patient inclusion. IM, intermediate metaboliser; PM; poor metaboliser; TG, tioguanine; TPMT, thiopurine S-methyl transferase.

In total, 502 requests for genotyping the *TPMT* gene were registered, which led to determination of the *TPMT* gene in 485 unique patients (Figure 2). Of these patients, 50 patients (10.3%) were intermediate TPMT metabolisers and 4 patients (0.8%) were poor TPMT metabolisers. Of all 54 patients with an aberrant *TPMT* genotype, 12 intermediate and 4 poor TPMT metabolisers were treated with TG in varying doses.

Of these 16 patients, 9 patients (56%) had CD and 13 patients (81%) were female, with a median age of 46 years (IQR 23–57) and median BMI of 24.8 kg/m^2^ (IQR 23.2–25.9). All baseline characteristics are shown in Table 1.

### 3.2. Poor Metabolisers—Clinical Observations

Of all four patients with a PM phenotype, three were initially treated with TG in a dose of 10 mg o.d., despite previously determined dysfunctional *TPMT* in two of these cases. These cases are briefly described below.

#### 3.2.1. Case 1

**Figure 3:**
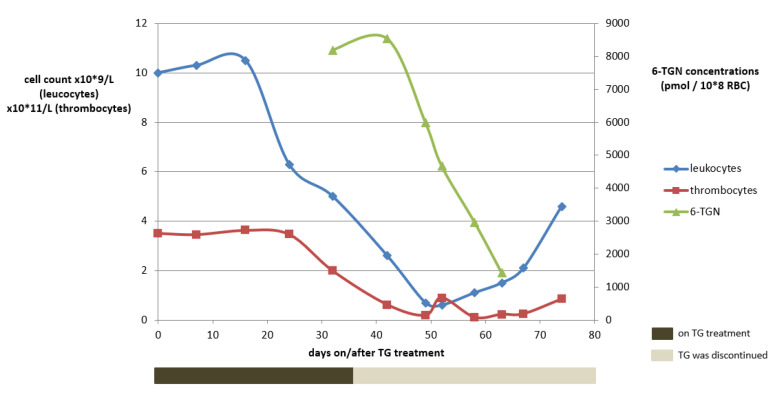
The course of white blood cell count abnormalities development in relation to the tioguanine use and the 6-TGN concentrations for case 1. Reference concentrations: 4–10 × 10^9^/L for leukocytes, 150–400 × 10^9^/L for thrombocytes and 250–900 pmol/8 × 10^8^ RBC for tioguanine. 6-TGN, 6-thioguaninenucleotides; RBC, red blood cells; TG, tioguanine.

A 48-year-old woman (73 kg) with a homozygous variant of the *TPMT* gene (*3A/*3A) suffering from chronic active UC was unsuccessfully treated before with infliximab (IFX) and vedolizumab. Treatment with TG was initiated in a dose of 10 mg o.d. No laboratory abnormalities were observed within the first month and clinical remission was achieved. At week four, 6-TGN concentrations were routinely measured, being as high as 8192 pmol/8 × 10^8^ RBC (reference value: <1000 pmol/8 × 10^8^ RBC). Meanwhile, haematological variables remained within the reference range (Figure 3).

Considering the potential cytotoxic 6-TGN concentrations, TG was immediately discontinued. Apart from headache, which was ascribed to (prior documented) migraine, no adverse drug events were reported until one week later, when nausea and dizziness occurred while headaches considerably increased. Two weeks after TG discontinuation, however, haematological toxicity increased, eventually comprising pancytopenia (grade IV according to the Common Terminology Criteria for Adverse Events (CTCAE)), with a leukocyte count of 0.9 × 10^9^/L (ref: 4–10 × 10^9^/L), an erythrocyte count of 2.3 × 10^12^/L (ref: 4–5 × 10^12^/L) and a platelet count of 62 × 10^9^/L (ref: 150–400 × 10^9^/L). Four weeks after TG discontinuation, the patient was hospitalized due to neutropenic fever and increased infection parameters and subsequently received intravenous antibiotics. Recovery took several weeks. TG treatment was not restarted and the exacerbated therapy-refractory colitis was operated on at a later date.

#### 3.2.2. Case 2

A 25-year-old woman (66 kg) with UC was treated with AZA therapy 100 mg o.d. After three weeks of treatment, she developed myelotoxicity with a leukocyte count of 1.8 × 10^9^/L and neutrophil count of 0.88 × 10^9^/L (ref: 2–7 × 10^9^/L). No *TPMT* genotyping was performed prior to treatment. AZA was discontinued due to leukopenia and mesalazine and local corticosteroids were introduced. After two years, UC relapsed and TG 10 mg o.d. was initiated according to the local treatment protocol. After four weeks of TG treatment, UC symptoms had improved, and laboratory results, including a hemogram and liver enzymes, were normal. The patient only reported severe fatigue. After six weeks, thiopurine metabolite concentrations were routinely measured and highly elevated 6-TGN concentration of 3872 pmol/8 × 10^8^ RBC were found, implying an increased risk of myelotoxicity. Treatment with TG was temporarily discontinued and *TPMT* genotype was requested, resulting in a *3A/*3C genotype. Notably, no myelotoxicity was observed. Haematological, liver safety parameters and 6-TGN concentrations were frequently monitored with a calculated prolonged half-life of TG of 12 days, compared to a reported half-life of approximately 5 days (range 3–9) [33]. In order to regain therapeutic 6-TGN concentrations, it was recommended to discontinue TG for at least two months. Following decrease in 6-TGN concentrations within reference values, TG therapy was resumed in an reduced dosage of 10 mg every two weeks (3.6% of registered dose), resulting in therapeutic 6-TGN concentrations ranging between 300–550 pmol/8 × 10^8^ RBC and beneficial therapeutic response in combination with adalimumab therapy. After one year, TG was discontinued upon patient’s own request. She has remained in remission with adalimumab for several years now.

#### 3.2.3. Case 3

A 19-year-old woman with CD (63 kg) was treated with MP 25 mg o.d. and therapy was discontinued due to nausea and severe stomach aches within two weeks after initiation. *TPMT* genotyping was performed in retrospect, showing two null alleles of TPMT (*3A/c.702T > A). With subsequent TPMT phenotyping, it was demonstrated that she was a poor TPMT metaboliser. Measurement of 6-TGN concentrations after two weeks resulted in a concentration of 2792 pmol/8 × 10^8^ RBC, possibly inducing the gastrointestinal adverse drug events. No haematological abnormalities were observed. One year later, after failing ADA monotherapy, TG was initiated in a reduced dose of 10 mg every three days (18% of registered dose), considering her *TPMT* deficiency. On this dose regimen, however, she developed 6-TGN concentrations of 4052 pmol/8 × 10^8^ RBC after four weeks of treatment whereupon TG was discontinued. Again, leukocyte count remained within the references ranges and neutrophil count slightly decreased (1.97 × 10^9^/L) two weeks later. Then, she developed a fever, cold chills and general malaise. Follow-up of 6-TGN concentrations was performed every 2–3 weeks, until 6-TGN concentrations were within reference values. Afterwards, a rechallenge with TG was initiated, optimized with frequent 6-TGN measurement, eventually leading to a dose regimen of 5 mg every three weeks (1.3% of registered dose), on which she has been in remission for 10 months now.

#### 3.2.4. Case 4

A 23-year-old woman with CD (67 kg) was treated with an adjusted dose regimen of AZA 25 mg every three days due to a known homozygous (*3A/*9) *TPMT* deficiency. Two weeks after initiation, therapy was discontinued due to a skin rash. A rechallenge of AZA therapy failed due to recurrence of the adverse drug event. Next, TG was initiated in a dose regimen of 10 mg once a week (7.1% of registered dose), resulting in therapeutic 6-TGN concentrations and clinical remission.

### 3.3. Intermediate Metabolisers—Clinical Observations

The 12 intermediate TPMT metabolisers (median bodyweight 78 kg, IQR 76–79) were treated with TG and had a median treatment duration of 17 months (IQR 5–31). The median daily dose of was 5 mg (IQR 5–10), matching a median percentage of registered TG dose of 23% (IQR 21–42%). Patient characteristics and details on TG treatment and 6-TGN concentrations are shown in Table 1. Four patients (33%) have been in remission with TG therapy until present. Eight patients (67%) discontinued TG therapy due to joint pain (*n* = 2, 17%), refractory IBD (*n* = 2, 17%), patient’s own request (*n* = 2, 17%), clinical remission for more than three years (*n* = 1, 8%) and toxic 6-TGN concentrations of >4000 pmol/8 × 10^8^ RBC after four years of treatment (*n* = 1, 8%).

The latter patient, however, had been treated with TG 20 mg o.d. for UC without *TPMT* genotyping nor routine measurement of 6-TGN concentrations in the start-up period. Notably, this was the only patient in this cohort who started with TG as early as 2014. After four years, a 6-TGN concentration of >4000 pmol/8 × 10^8^ RBC was found and TG was immediately discontinued. Genotyping of *TPMT* was performed in retrospect in 2018, confirming a heterozygous *TPMT* genotype (*1/*3A). At the time, no adverse drug events were observed and haematological parameters were slightly decreased: leukocyte counts of 3.0 × 10^9^/L and neutrophil counts of 1.74 × 10^9^/L. Additional tests were performed, and no signs of NRH were observed.

## 4. Discussion

In this cohort, we investigated TG dose regimens and adverse drug events, exclusively focusing on IBD patients with a hetero- or homozygous *TPMT* deficiency. We described several cases in which we both highlighted the risks and opportunities of TG initiation in *TPMT* deficient patients.

The most important risk of TG in poor *TPMT* metabolisers is that it can eventually lead to a long-lasting pancytopenia, even in relatively low standard TG doses for IBD. One patient suddenly developed pancytopenia (CTCAE grade IV) eight weeks after initiation of TG therapy, with sequelae necessitating hospitalization, ascribed to extremely elevated cytotoxic 6-TGN concentrations.

Generally, 6-TGN steady-state concentrations are attained after four to six weeks [11,33]. However, high inter-individual variability is observed, indicating that therapeutic drug monitoring (TDM) may help fine-tuning of dosage. In TG treatment, 6-TGN concentrations up to 1000 pmol/8 × 10^8^ RBC are considered safe and effective [33]. Notably, this upper limit has been proposed because of associations between high 6-TGN concentrations and risk of NRH [28]. With regard to the effectiveness and risk of myelotoxicity, however, associations with 6-TGN concentrations in TG have not been well established [34,35]. In a recent study, however, a 6-TGN cut-off value >682 pmol/8 × 10^8^ RBC was proposed for higher odds of clinical effectiveness [19]. Notably, cytotoxic 6-TGN concentrations up to 8 times the upper limit of the therapeutic range for TG (approx. 8000 pmol/8 × 10^8^ RBC) were found within four weeks of TG treatment in one case due to TPMT deficiency.

Previously, two other cases of TG-induced pancytopenia with unfavourable clinical sequelae due to TPMT deficiency have been reported [36,37]. In a recent retrospective cohort study, it was concluded that *TPMT* genotyping prior to TG initiation and 6-TGN measurements are not necessary in clinical practice [38]. This conclusion is contrary to our findings. The previous study, however, was based on a large IBD population, mainly including normal and intermediate TPMT metabolisers. Based on our cohort, exclusively focusing on patients with an aberrant TPMT enzyme activity, it was indicated that pre-emptive *TPMT* genotyping may be helpful to adapt the initial dose of TG to prevent (severe) myelotoxicity in patients with impaired TPMT function.

In this cohort, standard or slightly decreased TG dosages were deliberately initiated in several poor TPMT metabolisers. In contrast to AZA and MP, clinicians are not always aware of the associated risks of, particularly, TG use and the recommended dose adaptations in case of a TPMT deficiency. Specifically for TG, with a less complex metabolism compared to AZA and MP, standard dosages may lead to a relative late occurrence of leukopenia in intermediate or poor TPMT metabolisers. This unfavourable and potentially life-threatening clinical situation may only be identified via pre-emptively genotyping *TPMT* or via 6-TGN measurement a few weeks after TG initiation.

The risk of myelotoxicity due to TG use, in contrast to conventional thiopurines, is often debated. Elevated 6-MMPR formation after one week of treatment has been reported as an important predictor for thiopurine-induced myelotoxicity [39]. The absence of 6-MMPR in TG treatment might therefore be of importance. Another proposed reason for a weaker association is the less prominent role of TPMT in TG metabolism [34]. In our cohort, however, toxic 6-TGN concentrations could be ascribed to non-functional, homozygous variants of the *TPMT* gene, resulting in very low/absent TPMT enzyme activity. TPMT methylates TG and 6-TGN into the inactive metabolites 6-MTG and 6-methylthioguanine monophosphate (6-MTGMP), respectively (Figure 1). The weaker association of TPMT enzyme activity and 6-TGN concentrations in TG with respect to AZA and MP has not been studied properly.

In contrast, we also described three poor TPMT metabolisers who eventually were successfully treated with strongly reduced TG dosages (1.3–7.1% of registered dose). Notably, TG dose optimization based on frequent 6-TGN concentration measurement and strict monitoring of laboratory safety parameters is highly recommended, if not mandatory, immediately after initiation [40]. In a previous case-report, a strongly reduced TG dosage (6% of registered dose) had also been successfully applied in a homozygous *TPMT* deficient patient with CD [40].

In our cohort, we also found 12 heterozygous TPMT deficient patients who were treated with TG. In most patients, dose reduction was performed either immediately after pre-emptive *TPMT* genotyping or based on routine measurement of the elevated 6-TGN concentrations after four to six weeks. Reduced TG dosages (i.e., 10 mg every 2–3 days) led to 6-TGN concentrations within the therapeutic window with achievement of clinical remission for several months to years in the majority of the described cases. In contrast, higher dose regimens of 20 mg o.d. led to dangerous situations in intermediate TPMT metabolisers, arguing the practical and clinical relevance for *TPMT* genotyping or at least 6-TGN metabolite measurement within the first weeks after initiation.

According to current guidelines, TG dose recommendations are solely based on malignant conditions comprising 50–80% of the registered dose for heterozygous TPMT deficient patients [22]. In our cohort of IBD patients with heterozygous TPMT enzyme activity, median TG dosages were 23% of the registered dose. Based on our findings, TG may be applied in even lower dosages (25–40% of registered dose) for IBD treatment in intermediate TPMT metabolisers to maximize safety while maintaining clinical remission and therapeutic 6-TGN concentrations.

Some remarks on our study design should be made. First, we presented a relatively small series of TG-using patients with aberrant TPMT metabolism. Since data were retrospectively collected, we could not report on therapeutic effects, such as biochemical or endoscopic remission rates. Another remark is the reported prevalence of 0.8% poor TPMT metabolisers, indicative of a selection bias. Hetero- and homozygous TPMT variants are known to occur in, respectively, 11% and 0.3% of the Caucasian population, resulting in intermediate enzyme activity and lack of activity in the latter [41,42]. Obvious as in patients with (unexplained) thiopurine-induced toxicity or supratherapeutic 6-TGN concentrations, *TPMT* genotype was determined in retrospect, a higher number of *TPMT* polymorphisms of the total population was reported. Lastly, *TPMT* genotype was used to screen for at-risk individuals treated with TG, rather than TPMT enzyme activity. Here, *TPMT* genotyping was performed on the most common variants (*2, *3A, *3B and *3C), together accounting for more than 95% of all TPMT variants. In addition, *8 and *9 variants were also assessed, since these variants lie within the same fragments of the analysis. However, patients with another rare TPMT variant could therefore have been missed composing this cohort. Depending on the facilities and protocols in different hospitals, either pre-emptive *TPMT* genotyping or pre-emptive measurement of TPMT enzyme activity could be recommended in order to reduce the chances of (severe) myelotoxicity.

Based on our clinical observations and corroborated by previous reports, we believe that poor TPMT metabolisers can also be safely treated with a greatly reduced TG dose regimen of approximately 1–7% of registered dose [36,37]. Additionally, we recommend a cautious approach for intermediate TPMT metabolisers, namely a TG initiation of 25–40% of the registered dose. Strict monitoring of haematological parameters is recommended, if not mandatory, in the first few months, considering the possible delayed onset of TG-induced pancytopenia. We suggest to perform TDM four weeks after treatment initiation with reduced dosages, taking into account the prolonged time taken to reach steady-state concentrations.

## 5. Conclusions

Diminished or absent TPMT enzyme activity leads to a pharmacokinetic shift of tioguanine metabolism into elevated 6-TGN formation, which can ultimately lead to relatively late and potentially life-threatening myelotoxicity. However, TG treatment with a (strongly) reduced dose regimen remains a safe and effective option in IBD patients with dysfunctional TPMT, where strict genetic, therapeutic drug and safety monitoring is required, if not mandatory, particularly in the first months of treatment.

## Figures and Tables

**Table 1:** Patient characteristics.

	TPMT Genotype	Sex	Age (Years)	Body-Weight (kg)	BMI (kg/m^2^)	IBD Disease Type	TG Daily Maintenance Dose (mg)	TG Daily Dose (mg/kg)	TG Dose Percentage of Registered Dose ^#^	TG Treatment Duration (Months)	Reason for TG Discontinuation	6-TGN Concentrations (pmol/8 × 10^8^ RBC)
Poor TPMT metabolisers	*3A/*3A	F	48	73	23.0	UC	10	0.14	46.7%	1	Severe pancytopenia	Range 729–8536
	*3A/*3C	F	25	66	24.2	UC	0.7	0.011	3.7%	12	Patient’s own request	Range 306–3872
	*3A/c.702T > A	F	19	63	21.0	CD	0.24	0.0038	1.3%	9	N.A.	Range 435–4051
	*3A/*9	F	23	67	25.2	CD	1.4	0.021	7.0%	5	N.A.	Range 333–529
Intermediate TPMT metabolisers	*1/*3A	F	23	76	23.5	CD	5	0.07	21.9%	11	Refractory disease	323
	*1/*9	F	55	70	24.8	CD	5	0.07	23.8%	2	Joint pain	N.A.
	*1/*3A	F	59	60	19.4	UC	5	0.08	27.8%	31	N.A.	511
	*1/*3A	F	48	79	24.9	CD	10	0.13	42.2%	30	N.A.	725
	*1/*3A	F	43	80	32.0	CD	3.33	0.04	13.9%	37	Clinical remission	574
	*1/*3C	F	22	82	25.9	CD	5	0.06	20.3%	23	N.A.	462
	*1/*3A	F	22	56	22.4	UC	20	0.36	119.0%	48	Toxic 6-TGN concentration	4065
	*1/*3A	F	55	78	24.9	UC	5	0.06	21.4%	4	Refractory disease	610
	*1/*3A	F	28	78	28.7	CD	10	0.13	42.7%	5	Joint pain and sun allergy	850
	*1/*2	M	66	79	28.3	CD	10	0.13	42.2%	10	N.A.	421
	*1/*3A	M	71	90	28.4	UC	5	0.06	18.5%	4	Patient’s own request	820
	*1/*3A	M	63	76	23.2	UC	5	0.07	21.9%	36	Patient’s own request	431

^#^ Registered dose of tioguanine: 0.3 mg/kg bodyweight, not exceeding 25 mg/day. List of abbreviations: 6-TGN, 6-thioguanine nucleotides; BMI, body mass index; CD, Crohn’s Disease; F, female; M, male; N.A., not applicable; RBC, red blood cells; TG, tioguanine; TPMT, Thiopurine S-Methyl Transferase; UC, ulcerative colitis.

## Data Availability

The data presented in this study are available on request from the corresponding author. The data are not publicly available due to privacy reasons.
